# A spatial-temporal study for the spread of dengue depending on climate factors in Pakistan (2006–2017)

**DOI:** 10.1186/s12889-020-08846-8

**Published:** 2020-06-25

**Authors:** Waqas Shabbir, Juergen Pilz, Amna Naeem

**Affiliations:** 1 Institute of Statistics, Alpen Adria University of Klagenfurt, University Street 65-67, Klagenfurt, 9020 Carinthia Austria; 2 Quaid-i-Azam University, Islamabad, 45000 Pakistan

**Keywords:** Dengue, virus, epidemiology, choropleth, rainfall, temperature, geographical information system (GIS), generalised linear modelling (GLM), Pakistan

## Abstract

**Background:**

In Pakistan, dengue fever has become a major concerning factor, given that it is a relatively new disease compared to malaria. The number of people affected by dengue fever has increased at least 10-fold in the last 15 years in specific areas of Pakistan. Therefore, it is necessary to analyse this disease to reduce or prevent the effects of dengue fever in the region.

**Methods:**

Geographical information system (GIS) maps are used to identify the intensity of the spread according to the count of affected people in our study area. Generalised linear modelling (GLM) is used to study the significance of factors associated with dengue fever.

**Results:**

The dengue virus is present throughout the year in specific areas of Pakistan. Karachi and Lahore are most significantly affected with cases in these two most populous cities of Pakistan reported every year. In the study period (2006–2017), 2011 was the most devastating year for Pakistan. Lahore recorded more than 17,000 confirmed cases with 290 deaths in a single year. The GLM analysis shows rainfall, the average maximum temperature, and hospitals to be significant factors in the prevalence of dengue fever.

**Conclusion:**

This study finds that Sindh and Khyber Pakhtunkhwa are two of the primarily vulnerable provinces for the spread of dengue fever. Punjab has observed sporadic increases in dengue fever cases. In Pakistan, dengue cases increase in the rainfall season, especially during monsoon season. Lack of proper hospitals and clinics are another major factor, and mobile hospitals are needed in remote hotspot regions often affected by dengue fever. Finally, improved sanitation systems in metropoles would facilitate reducing breeding grounds for *Aedes Aegypti* larvae.

## Background

The dengue virus is spread through the bite of a mosquito into the bodies of mammals including humans. This virus is spread from the mosquitos of the family *Aedes Aegypti,* and the dengue virus is transmitted through the bite of female mosquitos [1]. The lifespan of a dengue mosquito is short, but after the larvae emerge from the eggs, they spread the dengue virus from 4 to 10 days on average during their lifespan [2–4]. The most suitable temperature for the eggs is 30°C. The combination of high temperatures and rainfall provides a substantial rise in dengue outbreaks [5]. The symptoms of dengue fever include nausea, pain in the limbs and joints, severe headache, vomiting, and so on [1, 6]. The early symptoms of dengue are similar to common fever and headaches, becoming increasingly intense over time. Different types of dengue viruses exist, and four types have been identified so far, namely DENV1, DENV2, DENV3, and DENV4 [2].

The World Health Organization has projected that 2.5 billion people across the globe are at risk of becoming affected with dengue fever in all continents except Antarctica [1]. The incidence of dengue fever has increased by 30 times worldwide, in the last 50 years alone in more than 100 countries. South-East Asia and southwestern America are two primary hotspots where the dengue virus has affected substantial populations [7, 8]. Rapid globalisation in the trade and tourism sectors and mass displacements (migration) have had major implications on human lives because globalisation has the potential to spread the disease to other parts of the world. Climate change effects are found in multiple parts of the globe and are responsible for making temperatures in particular regions more suitable for breeding mosquito-borne diseases, such as dengue fever [9–12]. Although the dengue virus is a noncontagious disease, it can be imported by different means, including air transportation. A viraemic person can carry the virus from one region or country to another because it can be transmitted via other mosquitos who are infected by the viraemic person [13–15]. The rise in global travel patterns is also a cause of the spread of new dengue virus serotypes and genotypes along with the spread of existing ones [16–18].

Recent climate-based research and projections put Pakistan in the top 10 list of countries affected by climate change, and numerous research articles have linked climate change and dengue fever [19, 20]. Studies have found that rainfall, temperature, and humidity are associated with the prevalence of dengue fever [21–24]. In Pakistan, all types of climates can be found: tropical to semi-tropical areas, deserts, and relatively cold areas in the north. Dengue fever in Pakistan has changed in terms of survivability against multiple factors, and the data in more recent years have suggested that the intensity of dengue fever has shifted from mild to severe [25].

Pakistan comprises a total area of 796,095 km^2^ and has four provinces (Sindh, Khyber Pakhtunkhwa, Punjab, and Balochistan) and two federally administrated regions (Gilgit-Baltistan and Kashmir). Our study is focused on the three provinces of Sindh, Khyber Pakhtunkhwa, and Punjab. We investigate the spread of dengue in 41 cities across these three provinces. The study area is illustrated in Fig. 2. Punjab is the biggest province in terms of population in Pakistan, and Lahore is its capital. Lahore has a population of 12 million people. Karachi is by far the biggest city in Pakistan and is the capital of the province of Sindh. It has a total population of 20 million people. It also generates most of the revenue for Pakistan. Big cities have major complexities in terms of managing various departments, which is observed in Karachi where pollution (air and land) is the norm. Karachi has experienced regular power outages and the lack of proper waste disposal. Moreover, excess rainfall water and poor sanitation make it one of the worst maintained metropolitan areas in the world, and it is a breeding ground for mosquitos. Except for a few hospitals, not many have the capacity to cope with emergencies, as in the case of dengue fever. Peshawar is the capital of Khyber Pakhtunkhwa and has a population of 4 million people. Islamabad is the capital of Pakistan and has a population of one million inhabitants.

Pakistan is 30.37° north and 69.34° east in South Asia with a population of over 210 million. The dengue virus is present in tropical and subtropical regions of Pakistan because it is a hospitable environment for the virus [26–28]. Pakistan experiences heavy rainfalls in the monsoon season and an increase in dengue cases in that period [29]. Dengue cases in Pakistan first surfaced in 1994; however, it only gained attention in the mid-2000s when the number of cases began to rise to large numbers in the coastal city of Karachi [30].

In this paper, we discuss the data and methods used to conduct the analysis. In the later part of our study, we plot the data spatiotemporally for 2006 to 2017 in three provinces of Pakistan. Finally, in the last section, we present the conclusions and suitable suggestions.

## Methods

### Study Area

In this study, we mapped the intensity of dengue fever in 41 regions of Pakistan over a 12-year period using the choropleth (intensity) mapping technique. Our study is conducted in three provinces: Sindh, Khyber Pakhtunkhwa, and Punjab. The regions under study differ in terms of climate, elevation, population densities, and so on.

### Area Selection

The regions were selected based on their population densities and importance in terms of economic growth. The climates of the regions vary to a high extent. The province of Sindh is mostly hot, humid, and dry. In contrast, Punjab has a very cold winter with a hot summer but receives heavy precipitation in the monsoon season. Khyber Pakhtunkhwa has a mild climate compared to the other two provinces; it includes regions with significantly higher elevations.

### Data Collection

The data on dengue were collected through news publishing agencies and published articles [31–35]. Dengue case data were collected yearly for the period 2006 to 2017. Climate data were collected through NOAA, USA, and online weather monitoring service providers [36, 37]. The climatic factors include maximum temperature, minimum temperature, and rainfall. The data on literacy, population, sanitation, and hospitals were collected through the World Wide Web and published reports from nongovernmental organisations (NGOs) [38, 39]. 

### Geographical Information Systems

**Fig. 1:**
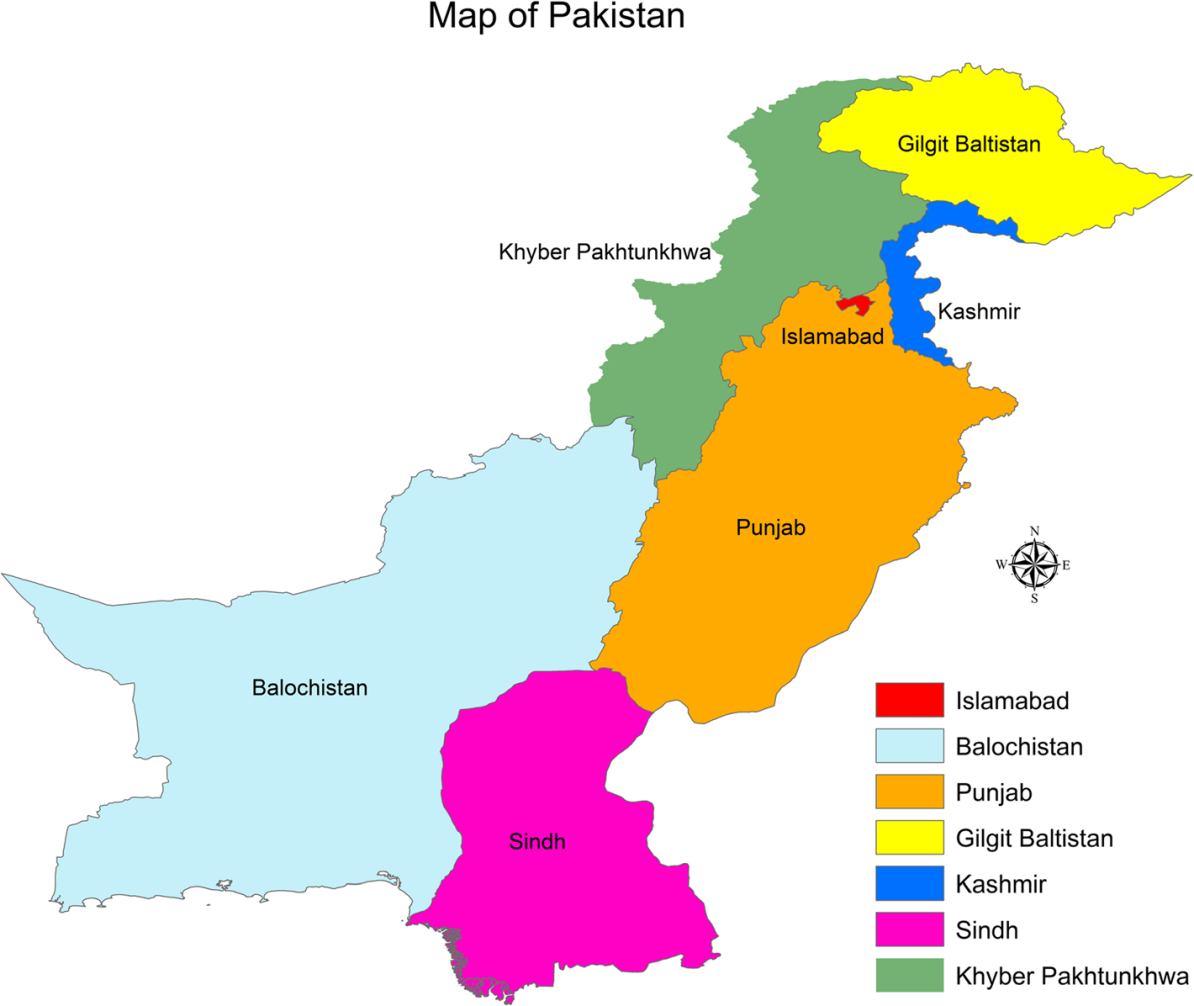
Provinces and administrated regions of Pakistan

**Fig. 2:**
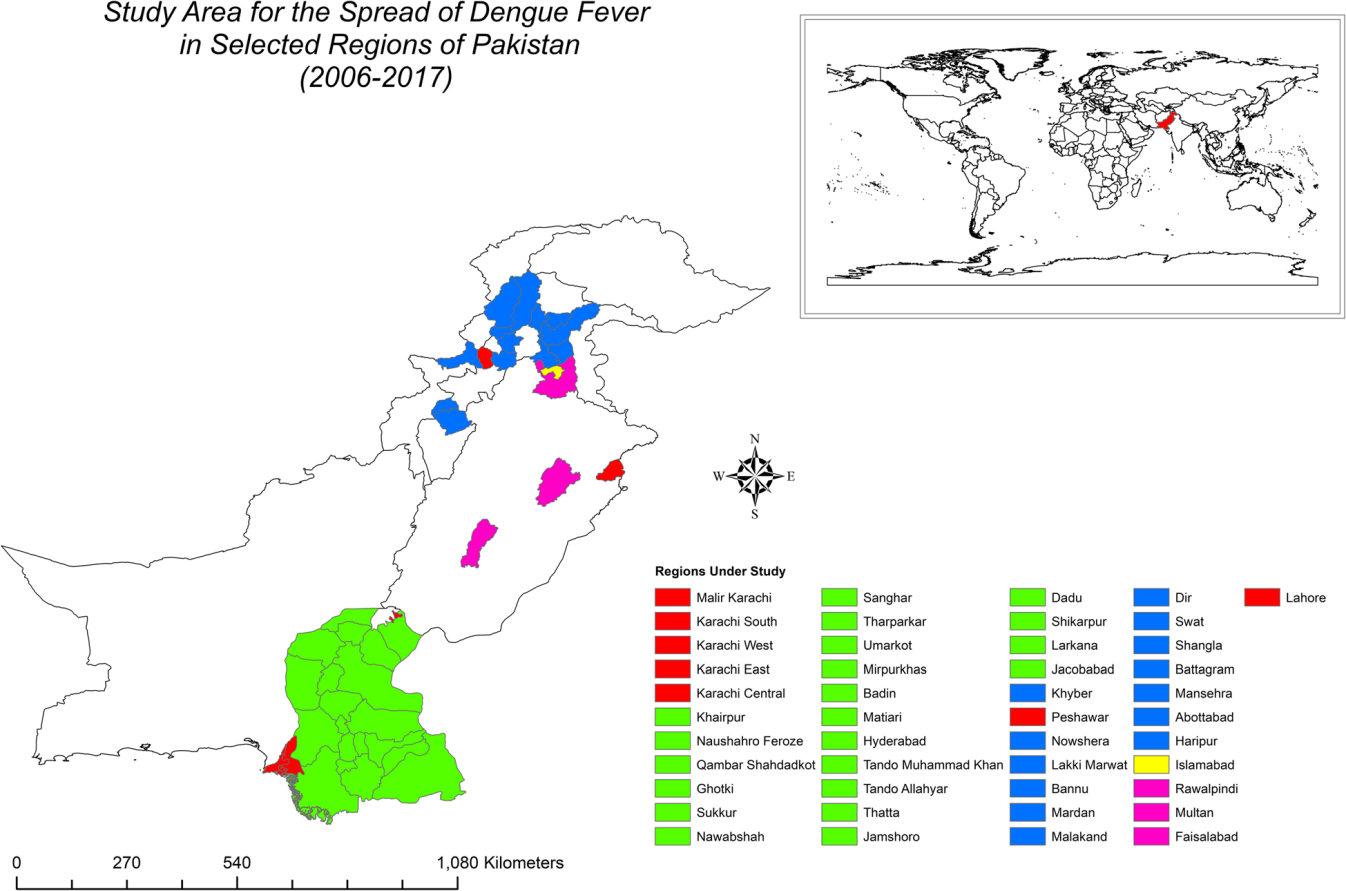
Study area: 41 cities selected from three provinces

Geographical information systems (GIS) are used for mapping quantitative data and are easy and well-defined tools to use when the aim is to demonstrate the intensity of an effect in specific areas to compare the area visibly because it is easier to observe which location exhibits more influence. ArcMap 10.5 is used as the GIS mapping tool for the dengue cases, population density, maximum and minimum temperatures, and elevation. Figure 1 illustrates the map of Pakistan, and the study area is depicted in Fig. 2.

### Data Analysis

The data were analysed using a GIS mapping technique. Approximately 86,000 dengue casualties (both deaths and number of cases) were observed in our preselected spatial units under study throughout the 12-year period. Most cases were reported in Lahore cumulatively, but this is primarily because of the anomaly observed in 2011 with the highest number of cases reported. The choropleth maps show the hotspots and areas that are less affected. We used different levels of intensities depending on the number of dengue fever cases in a given year. Generalised linear modelling (GLM) was applied to our climatic, social, and geographical factors involved in our dengue study. In addition, R-studio was used for the GLM analysis.

## Results

**Fig. 3:**
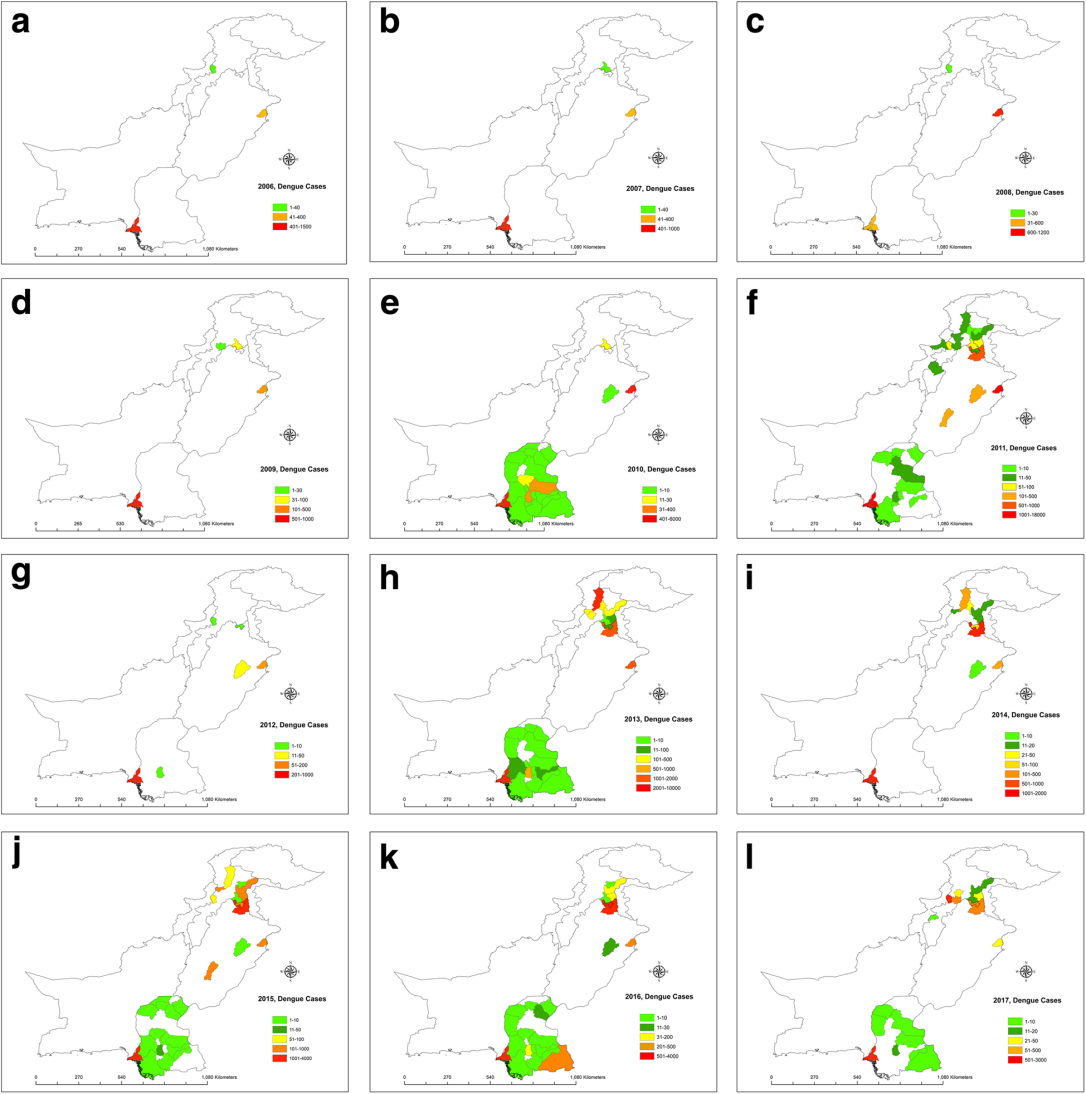
**a** Dengue incidences recorded in 2006. **b** Dengue incidences recorded in 2007. **c** Dengue incidences recorded in 2008. **d** Dengue incidences recorded in 2009. **e** Dengue incidences recorded in 2010. **f** Dengue incidences recorded in 2011. **g** Dengue incidences recorded in 2012. **h** Dengue incidences recorded in 2013. **i** Dengue incidences recorded in 2014. **j** Dengue incidences recorded in 2015. **k** Dengue incidences recorded in 2016. **l** Dengue incidences recorded in 2017

In this study, GIS was used to map the spatial and temporal intensity of dengue fever. In the figures depicted below, we mapped dengue fever cases according to different spatial units in our study area (Fig. 3 a-l).

The most affected years regarding dengue cases were 2011 and 2013 (for the study period 2006–2017) (Fig. 3 a-l). One possible reason that 2011 was the most devastating year is because of the heavy rainfall in the country causing heavy flooding; however, an increase in the number of dengue cases started to emerge prior to this in 2010. The study found that the main cause for higher than usual cases of dengue in Lahore could be attributed to the import of automobile tyres from Thailand (one of the hotspots for dengue in Asia) in 2011, and the tyres were infested with larvae contaminated with the dengue virus. Hence, the spread was massive [40].

The average temperatures in north-western Pakistan are mild, and these areas also record high amounts of rainfall. Northern Pakistan has several regions with high-altitude populations. However, the GLM estimates did not find elevation to be a significant factor affecting dengue fever cases, although we know that elevation influences the temperature. Our results using GLM indicate that the rainfall, average maximum temperature, and hospitals are also significant factors in the spread of dengue fever.

The availability of hospitals specifically equipped for dealing with dengue disease is a concern. People from remote areas in Sindh, Punjab, and Khyber Pakhtunkhwa usually must travel to big cities to be treated for this disease. The dengue virus inflicts additional stress on the existing healthcare system in specific seasons (anomalies such as higher than average rainfall, lower than average maximum temperatures, etc.). Therefore, it is necessary to provide mobile health facilities in remote areas.

**Fig. 4:**
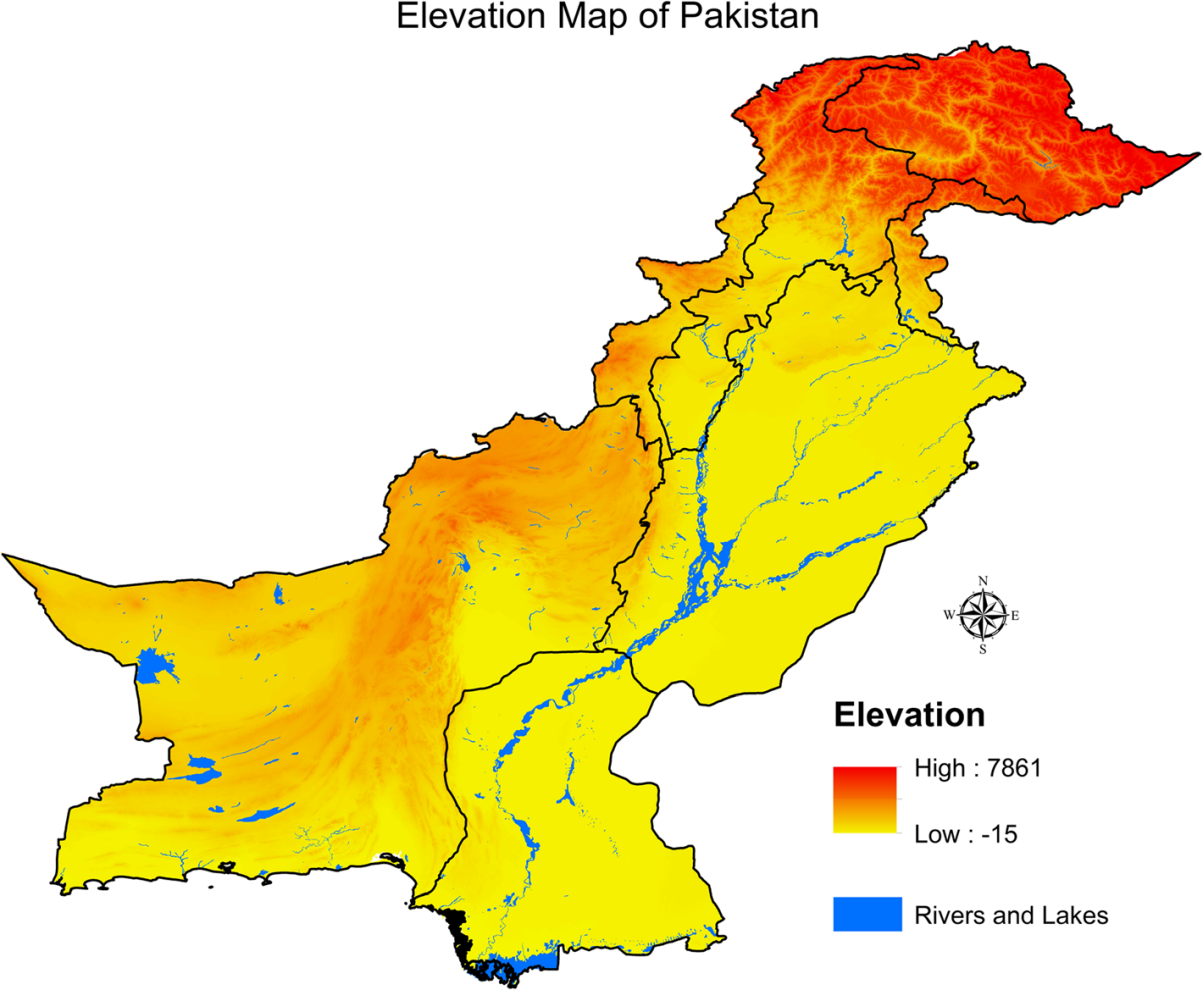
Elevation map of Pakistan

**Fig. 5:**
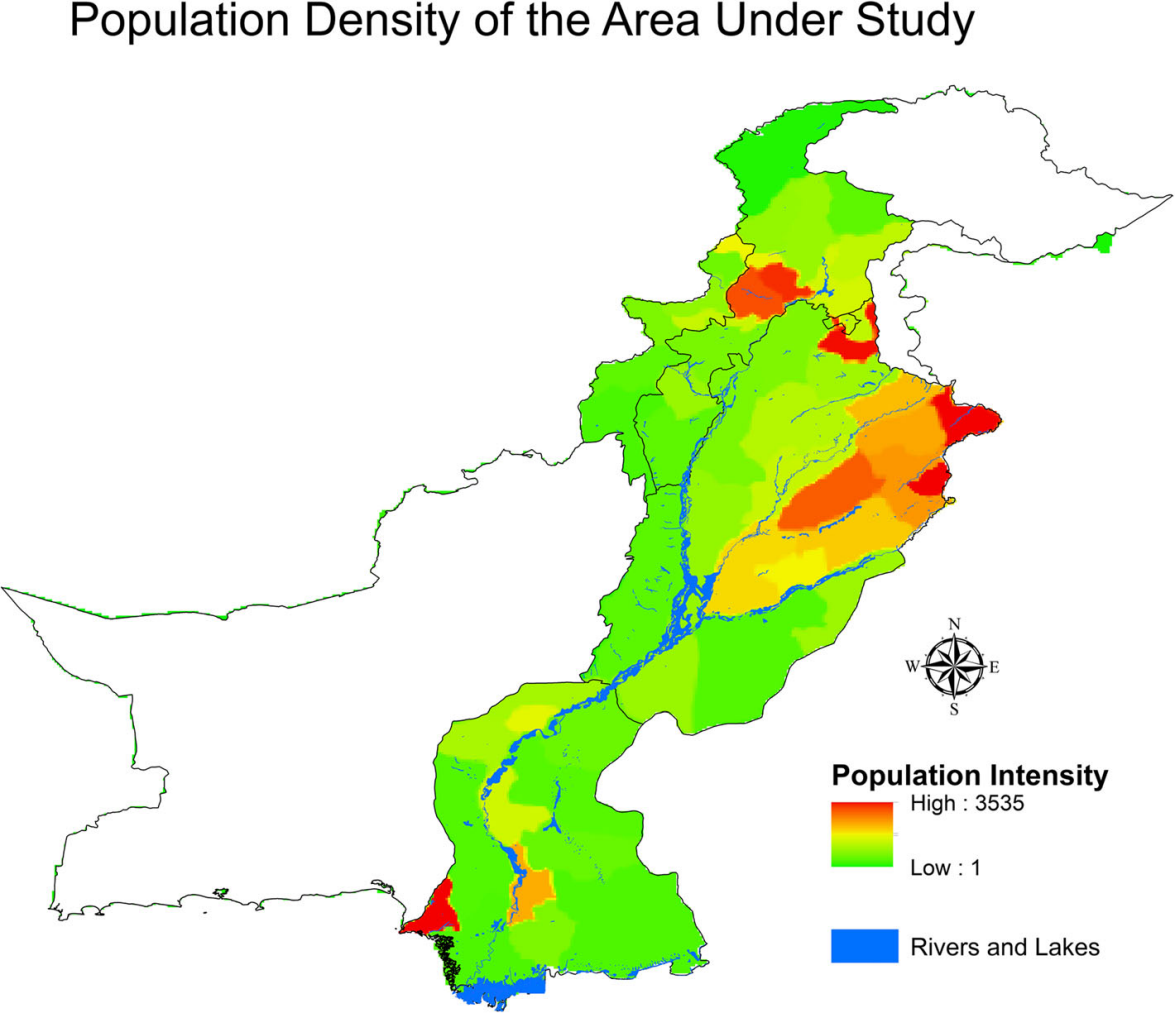
Population spread across Pakistan

Figures 4 and 5 illustrate the elevation map population densities in Pakistan. A high density of population lives in the lower elevation regions in the north-eastern region. Studies have found that a high risk of dengue incidence is associated with the population density (but not in all cases because the dengue virus is noncontagious), urbanisation, and the climate [41, 42].

**Fig. 6:**
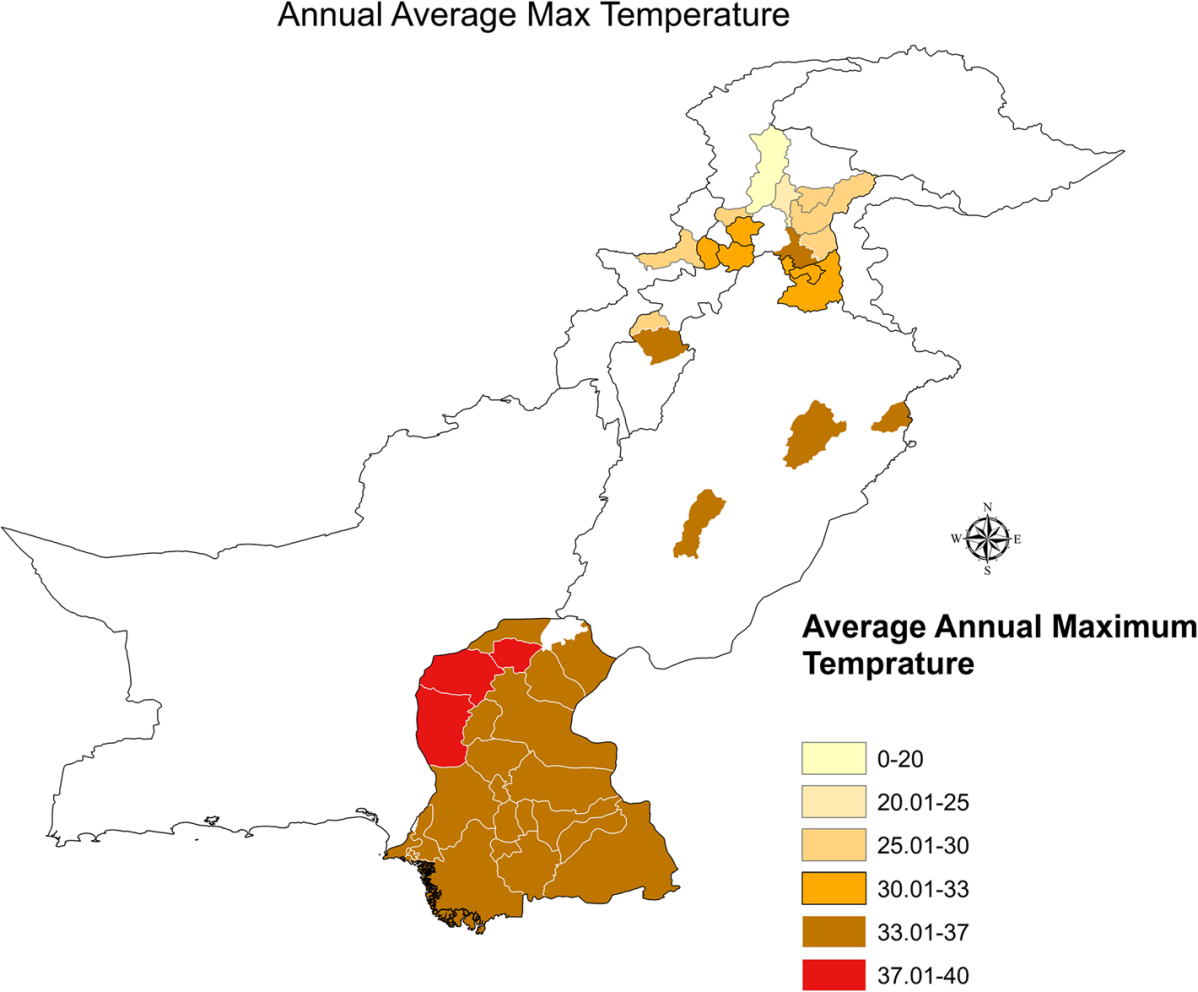
Annual average maximum temperature

**Fig. 7:**
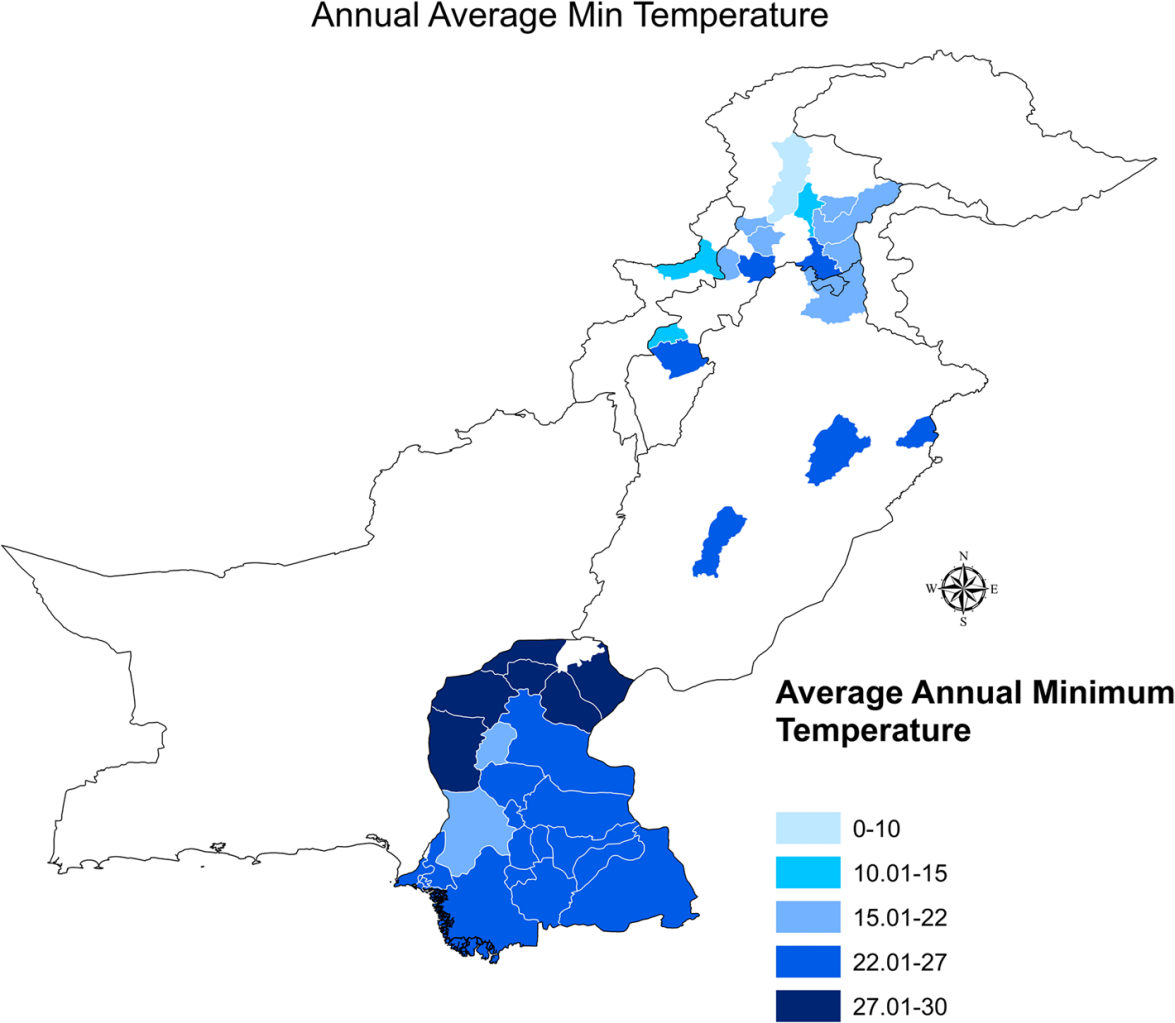
Annual average minimum temperature

We observed that numerous cases have arisen in cities with larger populations. The spread of dengue fever is attributed to several factors. Rainfall, average maximum temperature, and hospitals play a significant role in the prevalence of this disease, which was confirmed using GLM analysis. Figures 6 and 7 illustrate the annual average maximum and minimum temperatures in our study area.

We observed that southeast Pakistan is warmer compared to the northern and north-western regions of Pakistan and that temperature plays a key role in the spread of dengue fever because suitable temperatures for breeding larvae with the dengue virus are around 30°C. The southern cities of the provinces have the highest average minimum temperature, making these regions more vulnerable in the rainfall season, although the cycle of *Aedes Aegypti* is short-lived.

**Fig. 8:**
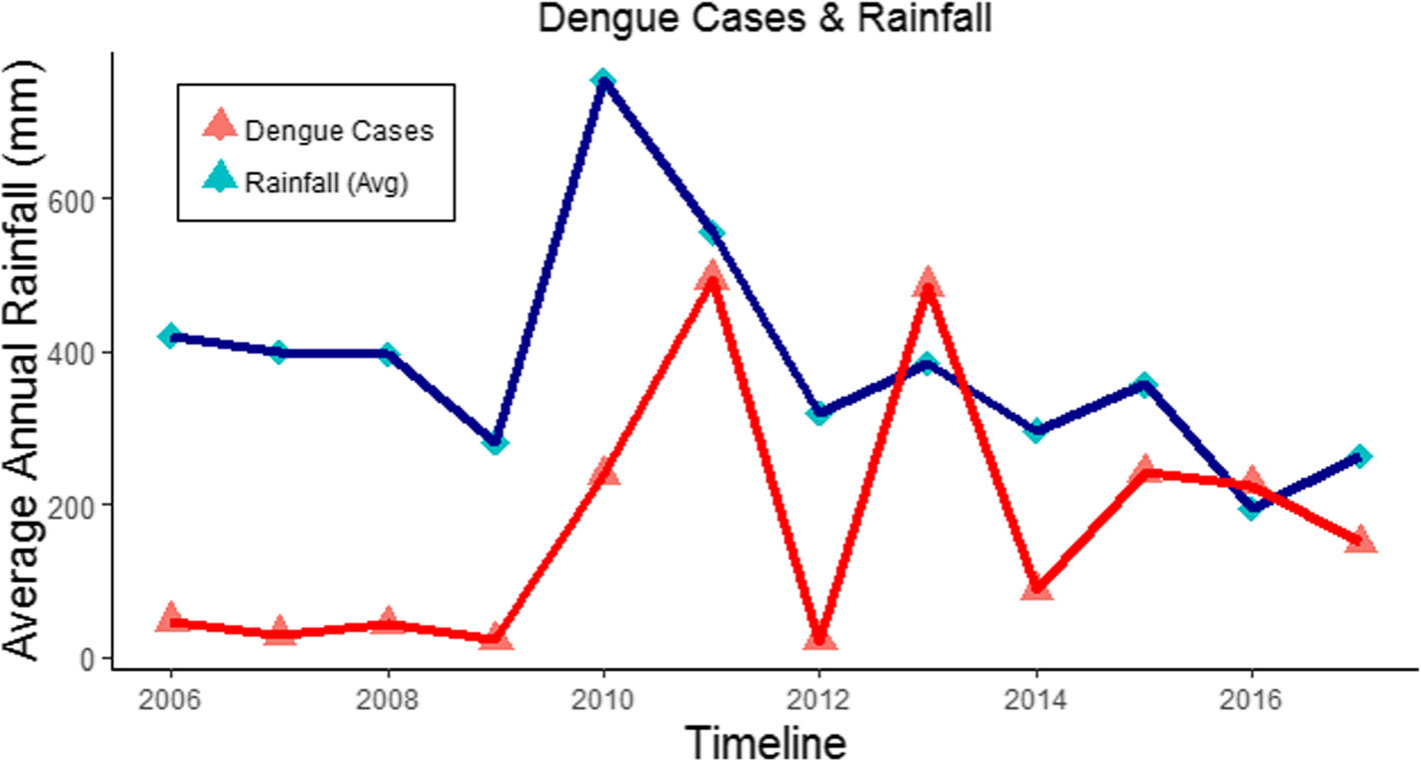
Dengue incidences and precipitation pattern

In Fig. 8, the number of cases and amount of rainfall are displayed. However, this does not conclusively (for all regions simultaneously) indicate that rainfall is the only factor causing the hike in dengue cases because some regions (in our study area) received more rainfall in the monsoon season than other regions in the same period. Our findings suggest that dengue cases tend to increase in erratic and increased rainfall seasons, along with other significant factors associated with the dengue virus.

In Table 1, an increase in the number of dengue cases occurred after an increase in rainfall. The historical annual average precipitation for the 41 cities is 424 mm. In the years 2010 and 2011, an accumulative increase of 54.38% in precipitation was recorded, compared to the historical average rainfall in this study, which was also the cause of major flooding across the country. Furthermore, in 2010, 2011, and 2013, significantly large numbers of dengue cases occurred. A decrease of more than 9% in the annual average rainfall in the study area occurred from 2006 to 2017; however, the provinces of Khyber Pakhtunkhwa and Sindh recorded heavier than average monsoon rains that killed or displaced hundreds of people in these two regions due to flooding. The excessive water in these regions is considered one reason for the numerous dengue fever cases. Other potential factors can be studied for a possible explanation of the spread of dengue from hot and humid locations (Sindh) to cold and mild locations (Khyber Pakhtunkhwa). Studies have shown that, regardless of dengue fever being directly noncontagious, it can spread from viraemic subjects from one place to another through infection by mosquitos [13, 16].

**Table 1:**
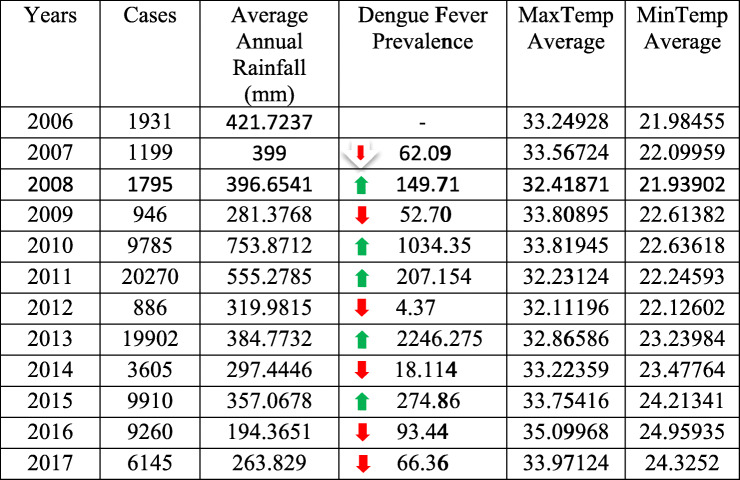
Dengue cases and environmental predictors (annual)

The variables included in the dengue study are rainfall, maximum temperature, minimum temperature, elevation, sanitation, population density, hospitals, and literacy. These variables were analysed using GLM to search for significant factors. The model is stated as follows:

1 $$ E\left(\mathit{\log}(y)\right)={\alpha}_0+{\alpha}_1\ast rainfall+{\alpha}_2\ast elevation+{\alpha}_3\ast maxTemp+{\alpha}_4\ast minTemp+{\alpha}_5\ast hospitals+{\alpha}_6\ast sanitation+{\alpha}_7\ast literacy+{\alpha}_8\ast popDensity, $$

where *y* is the number of dengue cases, and the GLM estimates are given in Table 2. A more detailed study can be found in [43].

**Table 2:** Generalised linear modelling estimates for the dengue study

Coefficients	Estimate	Std. Error	***t***-value	Pr(>|t|)
Intercept	1.002e+01	2.912e+00	3.442	0.00163 **
Rainfall	2.399e-03	1.174e-03	2.044	0.04928 *
Elevation	-1.669e-03	1.099e-03	-1.519	0.13867
Maxtemp	-3.510e-01	1.551e-01	-2.263	0.03054 *
Mintemp	1.717e-01	1.507e-01	1.139	0.26318
Hospitals	5.217e-01	1.873e-01	2.785	0.00893 **
Sanitation	2.715e-02	1.594e-02	1.704	0.09814
Literacy	1.882e-02	1.945e-02	0.968	0.34041
Popdensity	-3.468e-05	8.779e-05	-0.395	0.69543

Statistically significant data: ***0.001, **0.01, *0.05

## Conclusions

This study found that Sindh and Khyber Pakhtunkhwa are two main vulnerable areas for the spread of dengue fever. Punjab experienced the worst spread in 2011. Multiple factors are responsible for the spread and prevalence of dengue fever. Climate factors, such as rainfall and average maximum temperatures, play a significant role along with hospitals. The lack of proper hospital units is a highly significant factor in the spread of dengue fever. Areas most affected in Pakistan include areas that have large populations and higher population densities. The sanitation situation must be improved. Furthermore, awareness programmes are needed to help understand the dengue epidemic in remote and less developed regions of metropolitans. Preventive measures are needed that can be undertaken by the local governments in monsoon and rainy seasons to eradicate the breeding grounds for *Aedes Aegypti* larvae.

This manuscript has not been submitted in any other publishing source.

## Supplementary information

**Additional file 1.**

**Additional file 2.**

## Data Availability

All the data and material for this study are included in the manuscript, and additional data are provided in the supplementary material.

## Abbreviations

GIS: Geographical information systems
GLM: Generalised linear model
NGOs: Non-governmental organisations
NOAA: National Oceanic and Atmospheric Agency
